# Macular Thickness Assessed with Optical Coherence Tomography in Young Chinese Myopic Patients

**DOI:** 10.1155/2015/715798

**Published:** 2015-11-01

**Authors:** Minghui Zhao, Qiang Wu, Ping Hu, Lili Jia

**Affiliations:** Department of Ophthalmology, Shanghai Jiao Tong University Affiliated Sixth People's Hospital, Shanghai 200233, China

## Abstract

*Purpose.* To evaluate the variations in macular thickness in young Chinese myopic persons and the association with axial length (AL), spherical equivalence refraction (SE), age, intraocular pressure, and sex. *Methods.* In total, 133 young Chinese myopic subjects between 18 and 30 years of age were selected. The macular thickness was assessed using third-generation optical coherence tomography. AL, intraocular pressure, and SE were also measured. *Results.* The mean central foveal thickness was 191.1 ± 15.3 *µ*m. The macula was consistently thinner in women than in men. Central foveal thickness had a significant positive correlation with AL and a negative correlation with SE. In the inner and outer regions, the macular thickness had a positive correlation with SE and negative correlation with AL. *Conclusions.* The retina was thinner in women than in men. Associated with myopic progression and AL extension, the central foveal thickness increased, while the retinal thickness of the inner and outer regions decreased.

## 1. Introduction

Myopia is a public health problem in China and other countries in East Asia [1]. In high myopia patients (generally greater than −6.00 diopters (D)), scleral ectasias are relatively frequent and involve the posterior pole of the eye, leading to poor visual prognosis in adult life [2]. The risks of retinal detachment, chorioretinal atrophy, pigmentary degeneration, and posterior staphyloma also increase with severity of myopia and increase in axial length [3].

Previous histopathologic studies have found that myopia, especially high myopia, is associated with scleral increasing and retinal thinning. Optical coherence tomography (OCT) is a noninvasive, cross-sectional imaging technique that can measure macular thickness and is highly reproducible [4]. This technology allows* in vivo* measurement of retinal thickness to enhance the understanding of the pathophysiology of myopia and its relationship with the development of other ocular diseases.

A number of studies have reported the correlations between macular thickness and axial length or refractive error. However, most of those studies were performed in children, in adults over the age of 30 years, or in a wide range of age groups with one or mixed ethnicities [5–9]. Few studies investigated macular thickness values and the relationship with refractive error or axial length (AL) in young myopic Chinese patients aged 18 to 30 years.

The purpose of our study was to evaluate the variations in macular thickness in young Chinese myopic patients (aged 18–30 years) with different diopter (D) degrees and to assess the influences of axial length, refractive error, age, and sex using time domain-OCT (TD-OCT). These findings may contribute to knowledge regarding the macular thickness in the Han Chinese population.

## 2. Methods

The prospective study included 157 Chinese myopic subjects aged 18–30 years with various degrees of myopia who visited the Ophthalmology Department of Shanghai Jiao Tong University Affiliated Sixth People's Hospital between November 2012 and October 2013. To minimize selection bias, every third subject from the Physical Examination Center was chosen to participate. To eliminate any possible influence from different ethnic groups, only Han Chinese participants were selected. All subjects underwent a full ophthalmic examination, including determination of best-corrected vision acuity (BCVA), cycloplegic refraction, intraocular pressure (IOP) tested by a noncontact tonometer (Nidek, Gamagori, Japan), axial length measured by the IOL Master (Carl Zeiss Meditec, Inc., Dublin, CA, USA), and dilated fundus examinations. Pupillary dilation was induced by five cycles of 0.5% tropicamide (one drop), administered 5 min apart. The autorefractometer (ARK-700A, Nidek) was set to generate five readings of refraction 30 minutes after administration of the eye drops, and the median value given by the instrument was used for analysis. Inclusion criteria were as follows: age of 18–30 years, spherical equivalence refraction (SE) less than −0.50 D (SE was defined as spherical power plus half cylinder power), BCVA in each eye above 20/25, noncontact IOP between 10 and 21 mmHg, and no previous ocular disease or family history of glaucoma present. Subjects with concurrent diseases other than myopia, such as glaucoma, uveitis, media opacities, retinal diseases, or previous intraocular surgery, were excluded. Ethical approval for the study was obtained from the Shanghai Clinical Research Center. Informed consent was obtained from all study subjects. All work was conducted in accordance with the Declaration of Helsinki.

Macular retinal thickness was measured by a third-generation OCT (OCT-3, Carl Zeiss Meditec). The system (model 3000, software version B 3.0) permits cross-sectional imaging by acquiring a sequence of 128 interferometric axial reflectance profiles (A-scans) of the retina. The fast scan protocol completed total data acquisitions in 1.92 s. Pupils were dilated to at least 5 mm diameter during the OCT examination. The internal fixation target of the system was a large green asterisk on a red background. Scan length was adjusted to 6 mm before scanning. Six equally spaced intersecting radial scans through the center of the fovea were performed. Each radial scan comprised a circular area centered on the fovea. Three consecutive measurements were taken for each eye, and mean value was then calculated for each eye.

**Figure 1:**
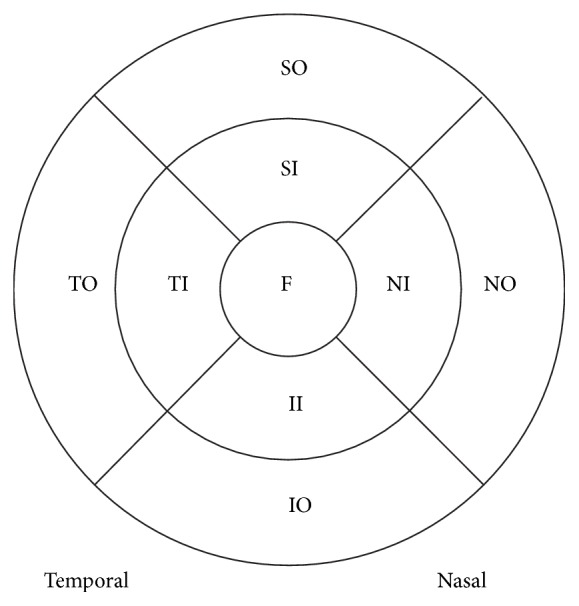
Macular map, automatically divided into nine Early Treatment Diabetic Retinopathy Study (ETDRS) sectors. F, foveola; TI, temporal inner sector; SI, superior inner sector; NI, nasal inner sector; II, inferior inner sector; TO, temporal outer sector; SO, superior outer sector; NO, nasal outer sector; and IO; inferior outer sector. Areas TI, SI, NI, and II form the inner region; areas TO, SO, NO, and IO form the outer region.

The mean retinal thicknesses were determined for nine sectors, as defined by the Early Treatment Diabetic Retinopathy Study (ETDRS) (Figure 1). The ETDRS areas included three concentric circles with diameters of 1, 3, and 6 mm; a central 1 mm circle represented the foveal area and inner and outer rings of 3 and 6 mm diameter, respectively. Each ring was divided into four quadrants: superior, nasal, inferior, and temporal. In this study, only the scans with signal strengths of at least six were analyzed. All measurements were taken by a single, trained examiner.

Statistical analysis was performed using the Statistical Package for Social Sciences (version 11.0; SPSS Inc., Chicago, IL, USA). Macular scans of the right eye were used for data analysis and presentation of the results. Descriptive statistics (e.g., count, mean, and standard deviation) were generated for all OCT outcomes and subject characteristics. The one-sample Kolmogorov-Smirnov test was used to test normal distribution. The intersex differences were assessed by independent-samples *t*-tests. Analysis of variance (ANOVA) with the Bonferroni post hoc test was used to compare mean thicknesses across the regions and quadrants in age, sex, AL, and SE groups. The associations between subject characteristics and macular thickness were evaluated using Pearson partial analysis. A *P* < 0.05 was defined as statistically significant.

## 3. Results

A total of 133 subjects were selected for analysis (72 women and 61 men). Twenty-four subjects were excluded due to OCT detection signal intensity < 6 (*n* = 12), alignment problems (*n* = 3), and IOP greater than 21 mmHg (*n* = 9). The mean age, axial length, SE, and IOP of the patients are shown in Table 1. There were no significant differences in mean age, axial length, and SE refraction between men and women (independent-samples *t*-test, *P* = 0.64, *P* = 0.51, and *P* = 0.81, resp.). However, the mean IOP was significantly higher in men than in women (independent-samples *t*-test, *P* < 0.01) (Table 1). Forty-seven eyes were low myopia (−0.50 D to −3.00 D), 57 eyes were moderate myopia (−3.00 D to −6.00 D), and 29 eyes were high myopia (<−6.00 D).

Macular thickness was normally distributed (Kolmogorov-Smirnov test). The central fovea was the thinnest of all the areas (mean thickness: 191.1 ± 17.3 *μ*m). The mean value of the inner circle was 268.4 ± 15.3 *μ*m, and the mean value of the outer circle was 236.7 ± 14.5 *μ*m. The mean retinal thickness of the whole macular region was 236.1 ± 16.3 *μ*m. Mean thickness varied across quadrants within the inner and outer regions. In the inner region, the superior quadrant was the thickest (271.4 ± 15.9 *μ*m), followed by that of the nasal (268.2 ± 17.3 *μ*m), inferior (265.7 ± 14.1 *μ*m), and temporal (256.4 ± 17.2 *μ*m) quadrants. In the outer region, the nasal quadrant was the thickest (258.6 ± 18.4 *μ*m), followed by the superior (240.8 ± 15.0 *μ*m), inferior (224.2 ± 13.6 *μ*m), and temporal quadrants (217.6 ± 13.9 *μ*m).

Table 2 shows the comparison of thickness parameters between men and women. Women showed significantly decreased retinal thickness in all ETDRS subfields except for superior, temporal, and inferior quadrants of outer regions, which did not show any significant difference. The macular measurements stratified by age are shown in Table 3. No statistically significant difference was found among age groups. Macular measurements among low, moderate, and high myopia patients in each ETDRS subfield are presented in Table 4. With the aggravation of myopia, the inner and outer region macular thicknesses were thinner, and the central macula was thicker. There were significant differences in all the quadrants of the ETDRS sectors among the three groups.

The relationships between foveal thickness and age, IOP, SE, and AL were analyzed by using Pearson partial correlation analyses (Table 5). No significant correlation was found between macular thickness and age or IOP for either sex (with adjustment for SE, IOP, and AL for the former, and adjustment for SE, age, and AL for the latter). Central foveal thickness had a significant positive correlation with AL (with adjustment for IOP, SE, age, and sex) and a negative correlation with SE (with adjustment for IOP, AL, age, and sex). In the inner and outer regions, the macular thickness had a positive correlation with SE (with adjustment for IOP, AL, age, and sex). AL was negatively correlated with the thickness of all the quadrants of inner and outer sectors, except the inner superior and nasal areas (with adjustment for IOP, SE, age, and sex).

## 4. Discussion

OCT uses infrared light with lower coherence interference measurement and can measure tissues and distances with good resolution. It is the best method to measure the retina thickness. Previous studies reported that spectral domain-OCT (SD-OCT) measurements resulted in a significantly thicker macular thickness measurement than the time domain-OCT (TD-OCT) [10–12]. The difference between the SD-OCT and TD-OCT measurements can be explained by the difference in the definition of retinal thickness. In TD-OCT, the posterior boundary is defined as the boundary of the inner segment/outer segment photoreceptor interface of the photoreceptor layer. In contrast, the retinal pigment epithelium is set as the posterior retinal boundary in Cirrus SD-OCT, while in the Spectralis SD-OCT Bruch's membrane is defined as the posterior boundary. In this study, we used TD-OCT, so we compared our results with published data using TD-OCT. The overall average macular thickness in this study (236.1 ± 16.3 *μ*m) compares favorably with the thickness reported in Wakitani et al. [13] (231 ± 15 *μ*m) and in Hee et al. [14] (230 ± 15 *μ*m).

We found that the central macula was the thinnest, followed by the outer region; the inner region was the thickest. These observations are consistent with the normal histological macular contours. Quadrant-specific variations were found in both the inner and outer regions, regardless of sex. The differences among inner region quadrants were smaller than those observed among the outer region quadrants, and the thickness in the nasal quadrant was significantly greater than the other three quadrants within the outer region. This finding is consistent with most studies [15–17] and could be explained by the anatomical relationship of the converging retinal nerve fibers with the optic disk. It is known that superior and inferior arcuate bundles of nerve fibers are crowded within the inner region and are relatively dispersed within the outer region, and the papillomacular bundle is more abundant in the outer nasal region, leading to the nasal quadrant being significantly thicker than the other three quadrants in the outer region [5].

We found that the thicknesses of the central macula, inner region, and nasal outer region were significantly greater in men than in women, in agreement with the findings of Al-Haddad et al. and Liu et al. [18, 19]. Two studies using SD-OCT reported no significant differences of macular thickness were found between men and women [4, 20]. However, the sample sizes of the two studies were both small, resulting in a relatively large error. The exact reasons for the intersex discrepancies are still unknown.

The relationship between retinal thickness and age has been widely reported. Lam et al. [21] did not find a relationship between retinal thickness and age. Song et al. [22] found that except for the central fovea, the thickness of the macular area decreased with increased age. The authors suggested that this thinning may be due to the loss of photoreceptor cells and ganglion cells, and the thinning of the retinal nerve fiber layer (RNFL) outside the central macula with aging. In this study, we found no correlation between age and macular thickness in all the quadrants of the ETDRS sectors for either sex. In our study, the subjects' age span was narrow (18–30 years), and this may have resulted in the deviation. More investigations are needed to determine the effects of age on macular thickness.

We found that young adults with longer AL have increased retinal thickness in the central fovea and decreased retinal thickness in the inner and outer regions, except in the inner superior and nasal areas. This correlated with the study of Lam et al., in which the minimum foveal thickness increased with axial length [21]. Hwang and Kim [23] also found that young myopic eyes showed thinner inner and outer macular thicknesses, and thicker central foveal thicknesses associated with longer AL. The reason for this finding is unknown, but a possible mechanism is that the increase in the AL of myopic eyes causes mechanical stretching of the sclera in the posterior pole, which would cause traction of the vitreous, and this may result in an increase of the fovea. The parafoveal region being more elastic undergoes stretching and peripheral thinning [24]. Thus, the increase in the foveal thickness with increasing degree of myopia can be an early sign of vitreoretinal traction, and the traction may be associated with retinal detachment, myopic traction maculopathy, and foveoschisis [21].

Our study showed that SE was negatively correlated with central fovea thickness and positively correlated with inner and outer macular thickness. This was similar to the study of Choi and Lee [25]. Ziylan et al. [26] compared the macular thicknesses of highly myopic children with healthy controls and found that foveal thickness was significantly greater in the high myopia group and parafoveal thicknesses of the inner and outer circles were significantly thinner in the high myopia group. However, in the studies of Barrio-Barrio et al. [6] and Al-Haddad et al. [27], no correlation between macular thickness and refraction was reported. However, in these two studies, patients with high refractive errors were excluded and this could have caused bias. It is known that the axial length increases with increasing degree of myopia, and elongation of the globe is associated with increasing central foveal thickness and decreasing foveal thickness in the inner and outer regions. Off-foveola fixation may also result in overestimation of foveal thickness. However, in our study, all subjects' BCVAs were better than 16/20. No pathological myopic changes were observed in fundus examination. The images obtained by the OCT were stabilized. For these reasons, we believe that the effect of off-foveola fixation was negligible.

In the present study, no significant correlation was found between IOP and macular parameters. The possible reason for this may be that macular thickness was affected only in the later stages of glaucoma and was less sensitive at the earlier stages of glaucoma and in healthy subjects [28].

In conclusion, this study described variations in macular thickness in myopic Han Chinese individuals, aged 18–30 years. We found that women had thinner retinal thicknesses than men, except in the superior, temporal, and inferior outer regions. None of the macular measurements displayed a significant correlation with age or IOP in either sex. Associated with myopic progression and AL extension, central foveal thickness increased, while retinal thicknesses of the inner and outer region decreased. These findings may improve interpretations of the results of OCT testing during diagnosis and management of ocular diseases in the young Han Chinese myopic population.

## Figures and Tables

**Table 1:** Baseline characteristics of patients.

	Age (year)	Axial length (mm)	Spherical equivalent (D)	Intraocular pressure (mmHg)
Women	23.9 ± 2.9	25.37 ± 1.04	−5.31 ± 2.13	16.27 ± 2.51
Men	24.6 ± 2.7	25.30 ± 1.01	−5.26 ± 2.07	15.81 ± 2.46
Total	24.4 ± 2.8	25.32 ± 1.02	−5.28 ± 2.10	16.00 ± 2.48
*P* value	0.64	0.51	0.81	<0.01

**Table 2:** Difference in macular measurements by sex.

Thickness	Women	Men	Total	*P*
(*µ*m)	(*n* = 72)	(*n* = 61)	(*n* = 133)	
Total	232.4 ± 16.7	238.7 ± 16.2	236.1 ± 16.3	<0.05
Central fovea	187.2 ± 17.2	193.4 ± 17.3	191.1 ± 17.3	<0.001
Inner region				
Average	265.3 ± 15.4	271.1 ± 15.4	268.4 ± 15.3	<0.001
Temporal	252.1 ± 16.3	259.7 ± 16.8	256.4 ± 17.2	<0.001
Superior	268.6 ± 15.6	274.1 ± 15.8	271.4 ± 15.9	<0.001
Nasal	265.4 ± 16.8	270.0 ± 17.5	268.2 ± 17.3	<0.001
Inferior	261.5 ± 14.6	268.6 ± 14.1	265.7 ± 14.1	<0.001
Outer region				
Average	235.9 ± 12.7	237.2 ± 12.2	236.7 ± 14.5	0.09
Temporal	216.4 ± 13.0	218.3 ± 13.7	217.6 ± 13.9	0.10
Superior	239.7 ± 14.6	241.6 ± 15.1	240.8 ± 15.0	0.34
Nasal	257.1 ± 17.7	260.5 ± 18.5	258.6 ± 18.4	<0.05
Inferior	224.2 ± 12.7	224.5 ± 13.9	224.2 ± 13.6	0.40

**Table 3:** Differences in macular measurements by age.

Thickness	18–20	21-22	23-24	25-26	27-28	29-30	*P*
(*µ*m)	(*n* = 18)	(*n* = 21)	(*n* = 20)	(*n* = 27)	(*n* = 26)	(*n* = 21)	
Total macula	235.2 ± 16.2	237.1 ± 15.1	235.8 ± 15.7	236.7 ± 16.0	234.8 ± 15.7	236.3 ± 16.4	0.71
Central fovea	191.7 ± 17.1	192.5 ± 18.2	193.3 ± 15.7	188.9 ± 17.6	190.4 ± 16.7	192.6 ± 17.0	0.23
Inner region							
Average	269.3 ± 13.1	268.4 ± 15.7	270.3 ± 14.5	267.6 ± 15.3	268.1 ± 17.1	268.0 ± 14.6	0.34
Temporal	255.9 ± 16.2	255.4 ± 17.1	256.3 ± 16.7	256.7 ± 17.6	256.3 ± 15.7	257.1 ± 18.0	0.53
Superior	272.0 ± 15.1	271.6 ± 16.4	270.3 ± 16.1	271.1 ± 16.9	270.8 ± 14.7	271.8 ± 15.0	0.64
Nasal	270.3 ± 17.4	268.3 ± 16.8	267.4 ± 19.2	268.0 ± 16.0	267.9 ± 16.3	268.2 ± 16.6	0.27
Inferior	266.4 ± 13.8	266.4 ± 13.9	265.2 ± 14.7	264.9 ± 14.0	265.1 ± 13.7	266.1 ± 15.1	0.13
Outer region							
Average	234.8 ± 13.4	236.1 ± 14.6	238.2 ± 15.0	236.9 ± 13.6	235.7 ± 14.3	237.4 ± 15.3	0.47
Temporal	219.8 ± 12.7	217.2 ± 13.8	216.4 ± 14.0	218.7 ± 14.5	216.6 ± 13.1	217.2 ± 15.1	0.43
Superior	239.6 ± 15.3	240.7 ± 14.4	241.7 ± 16.3	239.5 ± 13.4	241.1 ± 15.7	240.6 ± 14.1	0.62
Nasal	256.9 ± 16.1	258.7 ± 18.6	258.6 ± 17.4	261.1 ± 18.3	257.2 ± 18.9	258.3 ± 17.6	0.15
Inferior	223.7 ± 11.9	226.2 ± 13.7	226.1 ± 14.6	224.8 ± 14.3	223.1 ± 13.0	222.8 ± 14.8	0.12

**Table 4:** Differences in macular measurements by refraction.

Thickness	Low myopia	Moderate myopia	High myopia	*P*
(*µ*m)	(*n* = 47)	(*n* = 57)	(*n* = 29)	
Total macula	240.2 ± 14.6	237.4 ± 16.7	235.7 ± 16.4	<0.001
Central fovea	187.3 ± 15.1	190.5 ± 16.4	194.2 ± 17.9	<0.05
Inner region				
Average	269.6 ± 15.0	268.1 ± 14.6	268.3 ± 15.5	<0.05
Temporal	259.4 ± 16.6	255.7 ± 17.1	254.1 ± 16.8	<0.001
Superior	274.3 ± 15.0	271.1 ± 15.3	267.9 ± 16.2	<0.001
Nasal	271.2 ± 15.3	266.1 ± 16.9	266.1 ± 17.7	<0.001
Inferior	268.7 ± 13.7	263.6 ± 14.6	266.4 ± 12.8	<0.05
Outer region				
Average	239.5 ± 13.7	235.1 ± 14.2	233.8 ± 15.3	<0.001
Temporal	220.9 ± 13.1	215.2 ± 12.6	214.3 ± 14.7	<0.001
Superior	245.3 ± 10.6	240.4 ± 11.2	238.2 ± 12.7	<0.001
Nasal	260.9 ± 16.9	257.1 ± 17.1	254.6 ± 18.6	<0.001
Inferior	229.6 ± 13.1	224.7 ± 12.9	220.9 ± 14.6	<0.001

**Table 5:** Correlations between macular measurements and age, SE, AL, and IOP.

Thickness	Age		SE		AL		IOP	
(*µ*m)	*r* ^1^	*P*	*r* ^2^	*P*	*r* ^3^	*P*	*r* ^4^	*P*
Total macula	0.006	0.81	0.213	<0.001	−0.205	<0.001	0.003	0.91
Central fovea	0.031	0.59	−0.526	<0.05	0.418	<0.001	−0.037	0.60
Inner region								
Average	0.047	0.32	0.163	<0.05	−0.173	<0.05	0.011	0.83
Temporal	0.036	0.56	0.083	<0.05	−0.181	<0.001	0.015	0.51
Superior	0.021	0.35	0.139	<0.001	0.077	0.24	0.017	0.49
Nasal	0.065	0.61	0.097	<0.05	−0.014	0.37	0.003	0.86
Inferior	0.053	0.46	0.162	<0.001	−0.153	<0.05	0.002	0.92
Outer region								
Average	−0.038	0.49	0.302	<0.001	−0.216	<0.001	0.008	0.76
Temporal	−0.012	0.72	0.246	<0.001	−0.197	<0.05	0.003	0.81
Superior	−0.007	0.37	0.198	<0.001	−0.203	<0.001	0.015	0.67
Nasal	−0.064	0.69	0.383	<0.001	−0.184	<0.05	0.007	0.75
Inferior	−0.052	0.27	0.237	<0.001	−0.258	<0.001	0.001	0.84
